# Molecular insights into receptor binding of recent emerging SARS-CoV-2 variants

**DOI:** 10.1038/s41467-021-26401-w

**Published:** 2021-10-20

**Authors:** Pengcheng Han, Chao Su, Yanfang Zhang, Chongzhi Bai, Anqi Zheng, Chengpeng Qiao, Qing Wang, Sheng Niu, Qian Chen, Yuqin Zhang, Weiwei Li, Hanyi Liao, Jing Li, Zengyuan Zhang, Heecheol Cho, Mengsu Yang, Xiaoyu Rong, Yu Hu, Niu Huang, Jinghua Yan, Qihui Wang, Xin Zhao, George Fu Gao, Jianxun Qi

**Affiliations:** 1 CAS Key Laboratory of Pathogenic Microbiology and Immunology, Institute of Microbiology, Chinese Academy of Sciences, Beijing, 100101 China; 2 Department of Biomedical Engineering, Emory University, Atlanta, GA 30322 USA; 3 College of Life Science and Technology, Southeast University, NanJing, 210096 China; 4 Department of Biomedical Sciences, City University of Hong Kong, Hong Kong, 999077 China; 5 Laboratory of Protein Engineering and Vaccines, Tianjin Institute of Industrial Biotechnology, Chinese Academy of Sciences, Tianjin, 300308 China; 6 Shanxi Academy of Advanced Research and Innovation, Taiyuan, 030032 China; 7 Central Laboratory, Shanxi Province Hospital of Traditional Chinese Medicine, Taiyuan, 030012 China; 8 University of the Chinese Academy of Sciences, Beijing, 100049 China; 9 National Institute of Biological Sciences, Beijing, 102206 China; 10 College of Veterinary Medicine, Shanxi Agricultural University, Jinzhong, 030801 China; 11 Institutes of Physical Science and Information Technology, Anhui University, Hefei, 230601 China; 12 School of Laboratory Medicine and Life Science, Wenzhou Medical University, Wenzhou, 325035 China; 13 School of Life Sciences, Division of Life Sciences and Medicine, University of Science and Technology of China, Hefei, Anhui 230026 China; 14 Tsinghua Institute of Multidisciplinary Biomedical Research, Tsinghua University, Beijing, 102206 China; 15 CAS Key Laboratory of Microbial Physiological and Metabolic Engineering, Institute of Microbiology, Chinese Academy of Sciences, Beijing, 100101 China; 16 CAS Center for Influenza Research and Early-Warning (CASCIRE), Chinese Academy of Sciences, 100101 Beijing, China

**Keywords:** Viral proteins, SARS-CoV-2, X-ray crystallography

## Abstract

Multiple SARS-CoV-2 variants of concern (VOCs) have been emerging and some have been linked to an increase in case numbers globally. However, there is yet a lack of understanding of the molecular basis for the interactions between the human ACE2 (hACE2) receptor and these VOCs. Here we examined several VOCs including Alpha, Beta, and Gamma, and demonstrate that five variants receptor-binding domain (RBD) increased binding affinity for hACE2, and four variants pseudoviruses increased entry into susceptible cells. Crystal structures of hACE2-RBD complexes help identify the key residues facilitating changes in hACE2 binding affinity. Additionally, soluble hACE2 protein efficiently prevent most of the variants pseudoviruses. Our findings provide important molecular information and may help the development of novel therapeutic and prophylactic agents targeting these emerging mutants.

## Abstract (web summary)

The SARS-CoV-2 spike (S) protein mediates viral entry by binding of its receptor-binding domain (RBD) to the human angiotensin-converting enzyme 2 (ACE2) receptor and mutations of the S protein may have a great impact on virus transmissibility. Here, the authors characterize the interactions of six different SARS-CoV-2 RBD variants among them Alpha, Beta and Gamma and present crystal structures of these ACE2-RBD complexes.

## Introduction

Coronavirus disease 2019 (COVID-19) which is caused by SARS-CoV-2 has rapidly been declared as pandemic since its identification in late December 2019^1,2^. Up-to date, although tremendous efforts have led to the development of vaccine, combine with the preventive measures prescribed by the government around the world, the transmission of the virus among human are still going and numerous SARS-CoV-2 mutations with uncertain consequences for viral replication and transmission are being increasingly identified. The mutation of the spike (S) protein in SARS-CoV-2 has drawn wide concerns because S proteins mediate viral entry via their interaction with the angiotensin-converting enzyme 2 (ACE2) receptor^3,4^ and are the major target for vaccine development. SARS-CoV-2 variant D614G in the S protein rapidly become dominant around the world^5^. Recently, several novel SARS-CoV-2 variants of concern (VOCs) carrying D614G mutation have been linked to an increased number of infections at a global scale (Supplementary Fig. 1).

VOC Alpha (B.1.1.7 lineage or 501Y.V1) emerged in the United Kingdom (UK) in September 2020^6^. It has been shown to be 75% more transmissible^7^ with a 43–90% higher reproduction rate^8^ than earlier strains and has subsequently emerged as the dominant variant in the UK^7,8^. VOC Beta (B.1.351 lineage or 501Y.V2) was first identified in South Africa (SA) in October 2020, and has rapidly became the dominant strain in SA because of its high transmissibility^9^. The mutations of VOCs alter the viral fitness. The rapid spread of these variants of SARS-CoV-2 is likely due to the virus being more infectious, which means that small number virus particles can lead to infection. VOC Gamma (P.1 lineage or 501Y.V3) is a novel lineage tracing back to the B.1.1.28 lineage and was found to be circulating in Manaus, Brazil, in December 2020, with this isolate being identified in 42% of the specimens sequenced from this region in mid/late December^10^. And this Gamma variant was imported into Japan by travelers in early 2021^11^. N501Y in the receptor-binding domain (RBD) of S protein is a common substitution in Alpha, Beta, and Gamma strains, and is often found in combination with E484K and K417N (or K417T), with this combination described in the RBD of both the Beta and Gamma strains, respectively (Supplementary Fig. 1).

With the addition of variants circulating in humans, mutants have also been identified in susceptible farmed animals for fur. In Denmark, a SARS-CoV-2 variant referred to as the “Cluster 5” variant^12^, and designated as Mink-Y453F in this study, was identified in farmed minks and subsequently found to be transmissible between minks and humans^13^. Besides, both F486L and N501T mutations have been found in SARS-CoV-2 isolates from minks, as well as humans^14^, and these strains were designated as Mink-F486L and Mink-N501T in this study, respectively (Supplementary Fig. 1). Given that residues at positions 417, 453, 486, and 501 are involved in the interactions between the RBD and human ACE2 (hACE2)^4,15^, it is important to understand the detailed binding of these novel variant RBDs to hACE2.

Here, we examined six different SARS-CoV-2 RBD variants, including Alpha, Beta, Gamma, Mink-Y453F, Mink-F486L, and Mink-N501T. We found that these dominant mutations result in enhanced binding affinity to hACE2 receptor except for Mink-F486L, and that four variants pseudovirus particles except for Gamma and Mink-F486L show enhancement of viral entry to human cells. Analysis of molecular features of crystal structures revealed that replacements of key amino acids change bonding forces in the interaction interface. In addition, we demonstrate that the soluble hACE2 protein efficiently inhibit most of SARS-CoV-2 variants infections. Taken together, these data highlight the entry of new emerging variants and will provide a useful information to develop antiviral drugs against SARS-CoV-2.

## Results

### The binding characteristic of SARS-CoV-2 variants RBD to hACE2

**Fig. 1:**
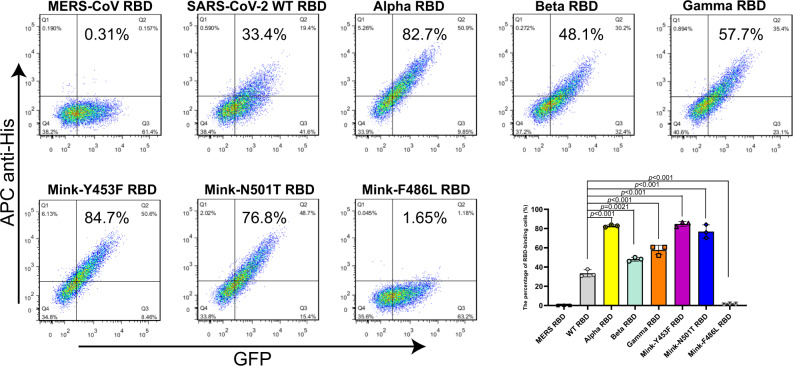
Characterization of binding between different RBDs and hACE2 using flow cytometry. BHK cells stably expressing GFP and hACE2 were incubated with His-tagged MERS-CoV RBD, SARS-CoV-2 WT RBD, Alpha RBD, Beta RBD, Gamma RBD, Mink-Y453F RBD, Mink-N501T RBD, and Mink-F486L RBD, respectively. APC anti-His antibodies were used to detect the His-tagged protein binding to the cells. Representative results from three experiments are shown. The mean ± SD percentages of RBD-binding cells in the three experiments are shown in the right lower bar chart. Statistical significance was analyzed using one-way ANOVA with a Tukey’s multiple comparison test for multiple groups.

The binding of RBD to the receptor hACE2 is an essential step for virus to entry cell, thus we first explored if the binding of six SARS-CoV-2 variant (including Alpha, Beta, Gamma, Mink-Y453F, Mink-F486L and Mink-N501T) RBDs to hACE2 were changed in comparison with wild-type (WT) SARS-CoV-2 (the first strain of SARS-CoV-2 which was isolated from a clinical patient on Jan 6, 2020, GISAID: EPI_ISL_402119) RBD. The SARS-CoV-2 WT RBD and variant RBDs were purified and then evaluated for hACE2 binding using flow cytometry (FACS) (Supplementary Fig. 2). SARS-CoV-2 Alpha, Beta, and Gamma, as well as Mink-Y453F and Mink-N501T RBDs, bound to the hACE2-expressing cells with higher affinity than WT RBD, with 33.4% positive cells (for WT RBD) versus 82.7% (for Alpha RBD), 48.1% (for Beta RBD), 57.7% (for Gamma RBD), 84.7% (for Mink-Y453F RBD), and 76.8% (for Mink-N501T RBD), respectively (Fig. 1). While Mink-F486L RBD bound to fewer hACE2-expressing cells with a 1.65% positivity rate (Fig. 1).

**Fig. 2:**
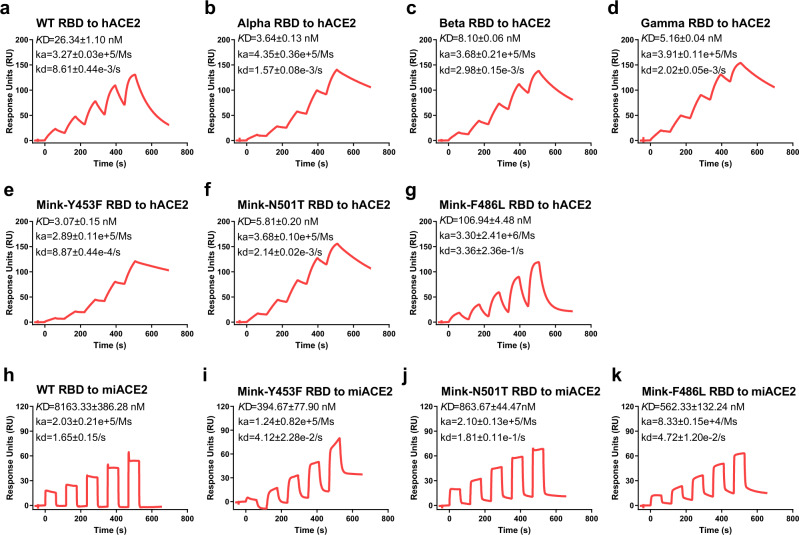
Binding affinity of SARS-CoV-2 RBDs to ACE2, characterized by SPR. **a**–**k** Mouse Fc (mFc)-fused hACE2 or miACE2 in the supernatant was captured in the CM5 chip via its interaction with the pre-immobilized anti-mFc antibody. Various concentrations of SARS-CoV-2 WT RBD (**a**), Alpha RBD (**b**), Beta RBD (**c**), Gamma RBD (**d**), Mink-Y453F RBD (**e**), Mink-N501T RBD (**f**), and Mink-F486L RBD (**g**) protein were used to evaluate their binding affinity for hACE2. Serially diluted WT RBD (**h**), Mink-Y453F RBD (**i**), Mink-N501T RBD (**j**), and Mink-F486L RBD (**k**) protein were measured the binding to miACE2. *K*_D_, ka, and kd values are all recorded and the representative results from three experiments are shown. The data are presented as the mean ± SEM of three independent replicates (*n* = 3).

To better understand the interactions between SARS-CoV-2 variant RBDs and hACE2, we measured their binding affinity using surface plasmon resonance (SPR). Mouse Fc (mFc) tagged hACE2 was captured in a CM5 chip that pre-immobilized with anti-mFc antibody, and the serially diluted variant RBDs were flowed through the chip. As shown in Fig. 2, SARS-CoV-2 WT RBD protein bound to hACE2 with an equilibrium dissociation constant (*K*_D_) of ~26.34 nM (Fig. 2a), which is similar to the previous results^16,17^. Alpha RBD, Beta RBD, Gamma RBD, Mink-Y453F RBD, and Mink-N501T RBD displayed higher affinities to hACE2 than WT RBD, with ~7, ~3, ~5, ~8, and ~4-fold increase in binding strength, respectively (Fig. 2b–f). Notably, both Beta RBD and Gamma RBD contain two more mutated residues than Alpha RBD, but displayed a little lower binding affinities for hACE2 than Alpha RBD. In order to understand which residues in Beta RBD and Gamma RBD contribute to the lower affinities, three single mutations (including K417N, K417T, and E484K), and three double mutations (containing N501Y/E484K, N501Y/K417N, and N501Y/K417T) were prepared and used to measure the binding affinities for hACE2. Both K417N and K417T mutations in RBD decreased ~2 fold affinity for hACE2, while E484K mutation exerted little effect (Supplementary Fig. 3). Similarly, both N501Y/K417N RBD and N501Y/K417T RBD also displayed ~2 fold lower affinity than N501Y RBD, but N501Y/E484K did not (Supplementary Fig. 3). Although both N501Y and N501T strengthened the interactions between RBD and hACE2, N501Y exhibits higher affinity than N501T. In contrast, Mink-F486L RBD bound to hACE2 with ~4-fold lower binding affinity than WT RBD (Fig. 2g). These results were consistent with the FACS assays. In a word, five variant (Alpha, Beta, Gamma, Mink-Y453F, and Mink-N501T) RBDs but not Mink-F486L RBD, increased association with hACE2.

Current data indicate that Mink-Y453F, Mink-N501T, and Mink-F486L emerged in farmed minks, when we evaluated their RBDs binding affinity for mink ACE2 (miACE2, Neovison vison^13,18^), we noted that WT RBD bound to miACE2 with ~8.16 μM affinity, which was ~310-fold lower than the affinity of WT RBD to hACE2 (Fig. 2h). In addition, Mink-Y453F RBD, Mink-N501T RBD, and Mink-F486L RBD exhibited much higher binding affinity for miACE2 than WT RBD with ~21, ~9, and ~15 fold, respectively (Fig. 2i–k). This provides some molecular evidence to explain why the mutant strains efficiently transmitted among minks.

### The molecular basis of hACE2 bound to SARS-CoV-2 variants RBD

To further elucidate the molecular mechanism underlying the binding of these SARS-CoV-2 variant RBDs to hACE2, we prepared for RBD-hACE2 complexes and obtained five crystal structures, namely Alpha RBD-hACE2, Beta RBD-hACE2, Gamma RBD-hACE2, Mink-Y453F RBD-hACE2, and Mink-F486L RBD-hACE2 at a resolution of 2.9 Å, 2.6 Å, 2.8 Å, 2.4 Å, and 2.7 Å, respectively (Table S1). Each complex structure was comprised of one copy of the RBD-hACE2 molecule in one asymmetric unit. The overall structure of Alpha RBD-hACE2, Beta RBD-hACE2, Gamma RBD-hACE2, Mink-Y453F RBD-hACE2, and Mink-F486L RBD-hACE2 were similar to the WT RBD-hACE2 structure, with a root-mean-square deviation (RMSD) of 0.196 Å (for 736 Cα atoms), 0.109 Å (for 725 Cα atoms), 0.198 Å (for 743 Cα atoms), 0.158 Å (for 712 Cα atoms) and 0.150 Å (for 763 Cα atoms), respectively (Supplementary Fig. 4), when compared with the WT RBD-hACE2 (PDB: 6LZG) structure.

**Fig. 3:**
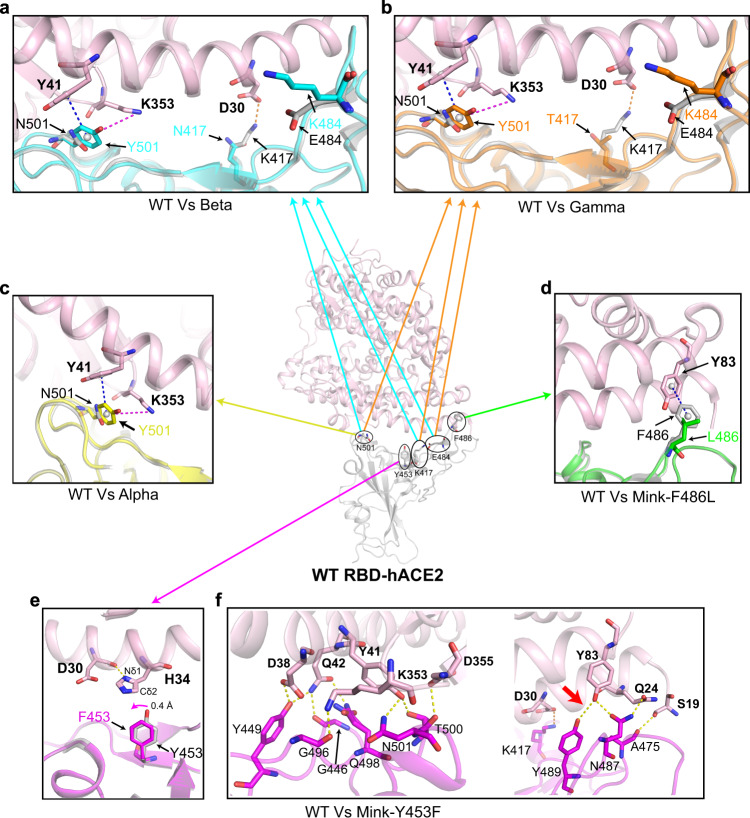
Structural comparison of WT RBD-hACE2 and each SARS-CoV-2 variants RBD-hACE2. **a**–**e** The WT RBD-hACE2 structure (PDB: 6LZG) is shown in the center. Superimposition of WT RBD-hACE2 and each variant RBD-hACE2 (including Beta RBD-hACE2 (**a**), Gamma RBD-hACE2 (**b**), Alpha RBD-hACE2 (**c**), Mink-F486L RBD-hACE2 (**d**), Mink-Y453F RBD-hACE2 (**e**)) are shown in each surrounding panel. In each structure the hACE2 is colored in light pink. SARS-CoV-2 WT RBD, Beta RBD, Gamma RBD, Alpha RBD, Mink-F486L RBD, and Mink-Y453F RBD are colored in gray, cyan, orange, yellow, green, and magenta, respectively. The key contact residues are shown as stick structures and labeled appropriately. The cation-π interaction, π-π stacking interaction, salt bridge, and hydrogen bonds are colored in magenta, blue, orange, and yellow, respectively. Hydrogen bond interactions were analyzed at a cutoff of 3.5 Å. **f** The detailed hydrogen bonds between the Mink-Y453F RBD and hACE2 are shown.

For the detailed mutated positions (Supplementary Fig. 5), in Alpha RBD-hACE2, Beta RBD-hACE2, and Gamma RBD-hACE2 structures, N501 is located in a loop structure and could be replaced by a Y without creating folding problems, it seems that the N501Y does not induce a large conformational change. Some very weak hydrogen bonds are possible between N501 and ACE2, but the phenyl of the Y501 side chain could make many new favorable nonbonded interactions with hACE2, i.e., a cation-π interaction with hACE2 K353 and a π-π stacking interaction with hACE2 Y41 (Fig. 3a–c). These noncovalent interactions in Y501 variant are stronger as compared to the WT type. Thus, Y501 significantly increased the interaction between RBD and hACE2 compared to N501, so the N501Y replacement in this region of the interface should be favorable for the interaction with hACE2. These results were consistent with cryo-electron microscopy structures of the N501Y SARS-CoV-2 spike protein in complex with ACE2^19^. In addition, the E484K substitutions would seem neutral or even unfavorable because it is far away from the RBD-interacting residues of hACE2 (Fig. 3a, b). This can also be confirmed by the results of the binding affinity of hACE2 with E484K single or double mutation constructions (Supplementary Fig. 3). Both K417N mutation in Beta RBD and K417T mutation in Gamma RBD destroyed the salt bridge formed by K417 and hACE2 D30 (Fig. 3a, b). Therefore, Beta RBD and Gamma RBD exhibited a higher binding affinity for hACE2 than WT RBD, but a little lower than Alpha RBD, consistent with the SPR results. In the Mink-F486L RBD-hACE2 structure, the mutation at F486L impairs the π-π stacking interaction formed by RBD F486 and hACE2 Y83 (Fig. 3d), resulting in the decreased interaction between Mink-F486L RBD and hACE2. We also predicted the structure of miACE2 and compared it with both Mink-F486L RBD-hACE2 and WT RBD-hACE2 structures. The residue T82 in miACE2 was clashed with the phenyl group of RBD F486 but not L486 (Supplementary Fig. 6). Thus, F486L mutation may be helpful for the interaction of SARS-CoV-2 RBD with miACE2.

In the Mink-Y453F RBD-hACE2 structure, the carbonyl group of hACE2 D30 forms a hydrogen bond with Nδ1 in hACE2 H34, leading to the Cδ2 from H34 forming a hydrophobic interaction with the phenyl group of RBD F453 (Fig. 3e). Thus, when compared with the hydrophilic hydroxyphenyl group of WT RBD Y453, the phenyl group at this residue (F453) shifts toward hACE2 H34 by 0.4 Å (Fig. 3e). In addition, the molecular dynamics (MD) simulations and molecular mechanics/Poisson-Boltzmann surface area (MM/PBSA) evaluations revealed that the binding energy of Mink-Y453F RBD-hACE2 was significantly more favorable than WT RBD-hACE2 (Supplementary Table 2), and F453 showed better binding energy than Y453 (ΔΔE_Binding_ = −1.03 kcal·mol^−1^), with the major contribution from the desolvation term (Supplementary Table 3). In addition, several adjacent residues were also shown to make favorable energy contributions to this binding when compared to the WT RBD-hACE2 complex, including residues R403 and K417 in the RBD, and D30 in the hACE2 (Supplementary Table 3). Moreover, the number of hydrogen bonds in the Mink-Y453F RBD-hACE2 increased, by one, when compared to WT RBD-hACE2. This includes hydrogen bond between hACE2 Y83 and Mink-Y453F RBD Y489 (Fig. 3f and Supplementary Fig. 7). Thus, the complex structure reported here provides the molecular explanation why Mink-Y453F RBD demonstrated an increased binding affinity for hACE2 when compared to WT RBD, which is observed in this and previous studies^20,21^.

### The transduction of SARS-CoV-2 variants pseudoviruses engaged by ACE2

**Fig. 4:**
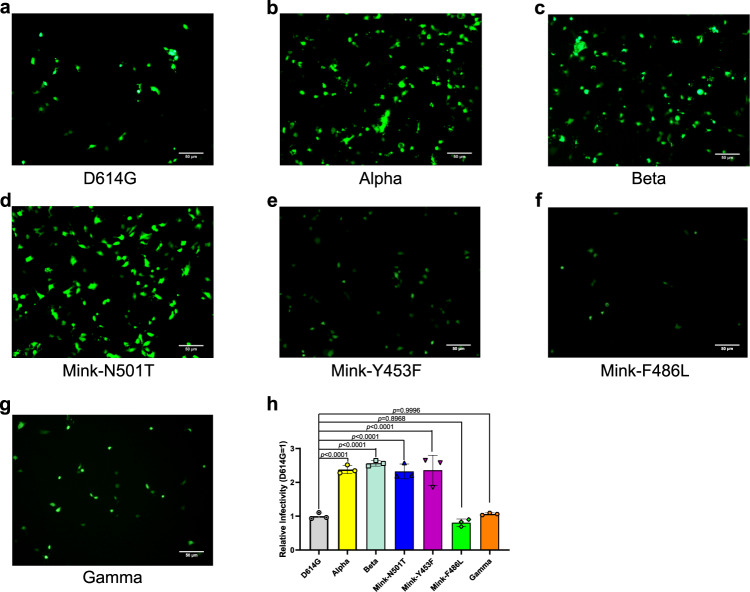
Entry of SARS-CoV-2 variant pseudoviruses into Huh7 cells. **a**–**g** The SARS-CoV-2 D614G (**a**), Alpha (**b**), Beta (**c**), Mink-N501T (**d**), Mink-Y453F (**e**), Mink-F486L (**f**), and Gamma (**g**) pseudoviruses’ entry into Huh7 cells as evidenced by GFP expression in transduced cells. The representative results from three experiments are shown. **h** The GFP-positive cells were quantified using FACS and representative results from three experiments are shown. The values indicate the mean of the three experiments and the bar suggest the SD. Relative infectivity was normalized against that of the D614G pseudovirus. Statistical significance was analyzed using a one-way ANOVA with Tukey’s multiple comparison test for multiple groups.

Considering that the binding affinity of SARS-CoV-2 variants RBD to hACE2 are changed, we further tested the potential influence of the SARS-CoV-2 variants on cellular infection using pseudovirus transduction. The same amount of pseudovirus that incorporate into the various SARS-CoV-2 variants S protein were infected hACE2-positive Huh7 cells and the GFP of pseudovirus was quantified by FACS for the transduction efficiency. As we saw in the binding affinity assays, pseudovirus particles of Alpha, Beta, Mink-N501T, and Mink-Y453F, but not Mink-F486L, showed increased transduction efficiency when compared to the D614G strain in Huh7 cells (Fig. 4a–f, and h). However, inconsistent with binding affinity, Gamma pseudovirus displayed the similar transduction efficiency with the D614G pseudovirus (Fig. 4g, h). In order to exclude the influence of binding of RBD to hACE2 on the different transduction efficiency between Beta and Gamma, N417T mutation was introduced to Beta S protein, which was designated as Beta-N417T that contains the same RBD sequence as Gamma. The transduction efficiency of Beta-N417T pseudovirus was similar to Beta pseudovirus and higher than Gamma pseudovirus (Supplementary Fig. 8). It suggested that other substitutions outside of the RBD also contribute to the change in transduction efficiency. Taken together, four out of six variants exhibited increased transduction efficiency.

### The neutralization of SARS-CoV-2 variants by soluble hACE2

**Fig. 5:**
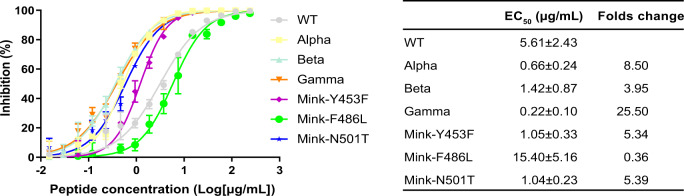
Neutralization of SARS-CoV-2 variants by hACE2. The EC_50_ values and folds change values are shown on the right panel. The folds change was normalized to WT, and the EC_50_ values are presented as the mean ± SEM of three independent replicates (*n* = 3). The representative results from three experiments are shown.

Considering that both Beta and Gamma were resistant to the antibodies-mediated neutralization^22,23^, soluble hACE2 was a potential therapy for COVID-19, especially under the background of higher affinity of SARS-CoV-2 variants to hACE2. We then tested the neutralization of soluble hACE2 protein to SARS-CoV-2 variants, and observed that soluble hACE2 protein efficiently prevent SARS-CoV-2 variants pseudoviruses from the entry into susceptible cells, and the inhibitory effect of soluble hACE2 protein to the SARS-CoV-2 variant pseudoviruses, with the exception of Mink-F486L, is higher than the SARS-CoV-2, especially Gamma (Fig. 5). It highlighted the importance of hACE2 targeted SARS-CoV-2 variants.

## Discussion

The deep mutational scanning suggests that single residue mutation K417N or K417T is likely to have minimal effect on the binding to hACE2, and that the E484K mutation may predictably enhance the binding^21^, while in this study the structures of Beta RBD-hACE2 and Gamma RBD-hACE2 indicated the mutations, K417N and K417T, decreased the RBD-hACE2 interactions while the mutation E484K displayed little impact. The structural information was confirmed by the SPR results that the single mutation (K417N or K417T) or double mutation (N501Y/K417N or N501Y/K417T) RBD displayed a lower binding affinity for hACE2 than WT RBD or RBD N501Y, but E484K or N501Y/E484K did not. Interestingly, the decreased impact caused by K417N or K417T could be quantitatively counteracted by the increased binding effect associated with the N501Y mutation.

Although we and other groups found a higher binding affinity of Mink-Y453F RBD to hACE2 compared with WT RBD^20,21^, it has been reported that the OH group of RBD Y453 is involved in direct interaction with hACE2 H34 in the cryo-electron microscopy (cryo-EM) complex structure of full-length hACE2 bound to RBD and B^0^AT1 (with the resolution of 2.9 Å)^24^. Interestingly, this interaction was not observed in the crystal complex structures of the hACE2 ectodomain bound to RBD (PDB: 6LZG and 6M0J) (with the resolution of 2.5 Å) or bound to Mink-Y453F RBD in this study (with the resolution of 2.4 Å). This discrepancy may be the result of the low resolution for hACE2 residue H34 within the RBD-hACE2-B^0^AT1 structure.

The changes in binding affinity and viral membrane fusion process may impact viral entry in the host. For instance, the N501Y mutation strengthens the binding affinity for mouse ACE2 and supports the adaptation of SARS-CoV-2 to mice in vitro and in vivo^25^. Corresponding with the binding affinity, more efficient transduction efficiency of pseudovirus particles of Alpha and Beta than D614G strain were observed in susceptive cells, although our assays were limited to pseudovirus viruses. It accounted at least in part for the higher transmissibility of Alpha^7^, and the fact that the frequency of viruses containing Alpha and Beta S sequences grew rapidly among the sequences available from the GISAID Initiative database (Supplementary Fig. 9a, b). In addition, the D614, which is outside RBD, located in the S1 subunit of the WT protein. The hydrogen bond formed by D614 residue and T859 residue in the adjacent propolymer S2 region disappeared after the mutation from D to G, which improved the cleavage efficiency of furin protease and made S1 protein more easily fall off from S2 protein, promoting the viral membrane fusion process, suggesting that D614G mutation increases entry efficiency^26^. Moreover, the P681R mutation in the spike protein, facilitates the spike protein cleavage and enhances the efficacy of viral fusion and further accelerates its speed of action^27^.

SARS-CoV-2 may acquire adaptive mutations that ensure efficient viral replication and transmission in other species, for example, by optimizing the interaction with host ACE2. The RBDs of three mink-origin variants: Mink-Y453F, Mink-F486L, and Mink-N501T, displayed a higher binding capacity to mink ACE2. These mutations may be adapted for the efficient use of mink ACE2 for entry. Thus, the number of both Mink-Y453F and Mink-F486L S sequences grew rapidly before the beginning of November 2020 (Supplementary Fig. 9c, d). However, Mink-N501T, whose binding affinity for hACE2, transduction efficiency, and frequency of S sequences (Supplementary Fig. 9e) were similar with both Alpha and Beta, was likely to adapt to transmission among humans, but Mink-F486L not. This may partly explain why Mink-F486L did not efficiently transmit to humans and suddenly disappeared following the implementation of the mink cull policy in the Netherlands and Denmark (Supplementary Fig. 9d). While, increasing human samples were detected to carry N501T (Supplementary Fig. 9e). However, Mink-N501T has not drawn enough attention. Considering SARS-CoV-2 has been detected in farmed minks in ten countries in Europe and North America^28^, our results suggest it should be evaluated in more detail in the future, especially in people who live or work on mink farms and are close to mink.

Although Mink-Y453F showed a higher level of transduction efficiency than Gamma, the frequency of their S sequences (GISAID) displayed a reverse tendency (Supplementary Fig. 9c, f). With the addition to the possibility of sampling bias and detection limitation in the relevant countries and regions, the reverse tendency suggested that other effects besides of viral entry brought out by the mutations, such as immune escape, probably impacted on the transmissibility of SARS-CoV-2 variants. It was supportively reported that Gamma escaped from vaccine-elicited neutralizing responses, but not Mink-Y453F^22^.

The result of live-virus neutralization assay showed that the Beta variant was poorly cross-neutralized by plasma from individuals with first-wave infections (did not contain the mutations associated with Beta) in South Africa^29^. The result of pseudoparticles infection indicated that entry of the Beta and Gamma variants were partially (Casirivimab) or fully (Bamlanivimab) resistant to antibodies used for COVID-19 treatment^23^. These results suggest that these variants may escape from neutralization by antibodies^23^ and convalescent plasma^29,30^. It is necessary to develop other therapeutic strategies for these variants. A potential drug-soluble human ACE2 protein was proved to reduce viral load in Vero E6 cells by 1000–5000-fold^31^, which has already conducted a two-part clinical phase 2 trial^32,33^. Soluble hACE2 protein displayed a more efficient inhibition to most SARS-CoV-2 variants, due to the higher binding affinity to hACE2. Thus, the engineered ACE2 that increased the binding affinity for SARS-CoV-2 could serve as a potential therapeutic against SARS-CoV-2 variants.

As more SARS-CoV-2 variants continue to emerge and the major SARS-CoV-2 variants continue to spread, characterization of the hACE2-binding affinity and transduction efficiency of SARS-CoV-2 variants will help us understand SARS-CoV-2 transmission. The molecular features of variant RBDs binding to hACE2 provides valuable information helping us understand the entry mechanism of SARS-CoV-2 variants and aiding in the development of novel vaccines and specific drugs that target the SARS-CoV-2 entry process.

## Methods

### Cells

Expi293F cells (Gibco) were cultured at 37 °C in SMM 293-TII Expression Medium with 5% CO_2_ in a shaking incubator (140 rpm). Sf9 cells (Invitrogen) and High Five cells (Invitrogen) were cultured at 27 °C in Insect-XPRESS medium (LONZA) in a shaking incubator (120∼130 rpm).

HEK293T (ATCC), BHK21 (ATCC) and Huh7 cells (3111C0001CCC000679) were cultured at 37 °C in Dulbecco’s Modified Eagle medium (DMEM) supplemented 10% fetal bovine serum (FBS) at 5% CO_2_.

### Gene cloning, expression and protein purification

The coding sequence of hACE2-mFc (residues 1-740, GenBank: NP_001358344) or miACE2-mFc (residues 1-740, GenBank: QPL12211) were cloned into pCAGGS vector (MiaoLingPlasmid). The plasmids were transiently transfected into HEK293T cells using PEI and then, 48 h later, the cell supernatants were collected, concentrated and used in the SPR assays.

The DNA sequence encoding hACE2 (residues 19-615, GenBank: NP_001358344) was inserted into the Baculovirus transfection vector pFastBac1 (Invitrogen) using the *Eco*RI and *Xho*I restriction sites. The gp67 signal peptide sequence was added to the N-terminus of the hACE2 gene for protein secretion, and the Hexa-His tag sequence was added to the C-terminus of the hACE2 sequence for protein purification. The hACE2 protein was expressed using the Bac-to-Bac Baculovirus expression system and used for crystallization. The pFastBac1-hACE2 plasmids were transformed into DH10Bac *E. coli* to produce recombinant bacmids. Transfection of the bacmids using FuGENE 6 Transfection Reagent (Promega) and virus amplification were carried out in Sf9 cells, and the proteins were expressed in High Five cells. The supernatants were collected 48 h post-infection.

The DNA sequences encoding hACE2 (residues 1-740, GenBank: NP_001358344) were cloned into the pCAGGS vector with Hexa-His tag at the C-terminus. The DNA sequences encoding SARS-CoV-2 WT RBD (spike residues 319-541, GISAID: EPI_ISL_402119) or MERS-CoV RBD (spike residues 367-606, GenBank: JX869050) were inserted into the pCAGGS vector with IL10 signal peptide sequence at the N-terminus and the Hexa-His tag at the C-terminus. The SARS-CoV-2 variant RBD plasmids (including Alpha RBD, Beta RBD, Gamma RBD, Mink-Y453F RBD, Mink-F486L RBD, Mink-N501T RBD, RBD N501Y/E484K, RBD N501Y/K417N, RBD N501Y/K417T, RBD K417N, RBD K417T, and RBD E484K) were constructed via site-directed mutagenesis using the Mut Express II Fast Mutagenesis Kit V2 (Vazyme). The recombinant RBD and hACE2 (used for pseudovirus neutralization assays) proteins were expressed in Expi293F cells after plasmid transfection using Sinofection Transfection Reagent (Sino Biological). The supernatants were collected 5 days post-transfection.

The supernatants containing hACE2 or RBD proteins were purified via affinity chromatography using a HisTrap HP 5 mL column (GE healthcare) and the target proteins were eluted in an elution buffer composed of 20 mM Tris (pH 8.0), 150 mM NaCl, and 300 mM imidazole. The samples were then purified using gel-filtration chromatography on a HiLoad 16/600 Superdex 200PG column (GE healthcare) in a buffer containing 20 mM Tris (pH 8.0) and 150 mM NaCl.

### Complex preparation and crystallization

Purified hACE2 and each SARS-CoV-2 variant RBD protein (including Alpha RBD, Beta RBD, Gamma RBD, Mink-Y453F RBD, and Mink-F486L RBD) were mixed and incubated on ice for 2 h. The mixture was then purified on HiLoad 16/600 Superdex 200PG column in a buffer containing 20 mM Tris (pH 8.0) and 50 mM NaCl. The SARS-CoV-2 variant RBD-hACE2 complex proteins were then concentrated to 15 mg/mL for crystallization. All crystallizations were performed using a vapor-diffusion sitting-drop method with 0.8 μL protein mixing with 0.8 μL reservoir solution at 18 °C. High-quality crystals for both the Beta RBD-hACE2 and Gamma RBD-hACE2 complexes were obtained when using 0.1 M MES (pH 6.5), 12% w/v PEG 20000 at a concentration of 15 mg/mL at 18 °C. Complex crystals of Alpha RBD-hACE2, Mink-Y453F RBD-hACE2, and Mink-F486L RBD-hACE2 were grown in 0.1 M MES (pH 6.5), 10% w/v PEG 5000 MME, 12% v/v1-Propanol at a concentration of 15 mg/mL at 18 °C.

### Data collection and structure determination

Prior to collecting diffraction data, all crystals were cryo-protected by briefly soaking in reservoir solution supplemented with 20% (v/v) glycerol and then flash-cooled in liquid nitrogen. All X-ray diffraction data were collected at Shanghai Synchrotron Radiation Facility (SSRF) BL17U. The datasets were indexed, integrated, and scaled using HKL2000^34^. The structures of variant RBD-hACE2 were determined via molecular replacement method using Phaser^35^ with the previously reported structures of SARS-CoV-2 RBD-hACE2 (PDB: 6LZG) as a search model. The atomic models were built using Coot 0.8.2^36^ and the refinements were completed using Phenix.refine^37^. MolProbity was used to assess the stereochemical quality of the final models^38^. The data collection, processing, and refinement statistics were summarized in Supplementary Table 1. All structural figures were generated using the PyMOL 4.5 software (https://pymol.org/2/).

### Surface plasmon resonance (SPR) assay

The SPR assays were performed to test the interactions between mFc-fused ACE2 (including hACE2 and miACE2) and SARS-CoV-2 variant RBDs using a BIAcore 8 K (GE Healthcare) with a CM5 chip (GE Healthcare) at 25 °C in single-cycle mode. SARS-CoV-2 WT RBD was used as a positive control. The buffer system was PBST (10 mM Na2HPO4, 2 mM KH2PO4, pH 7.4, 137 mM NaCl, 2.7 mM KCl, 0.005% Tween 20) and the anti-mIgG antibody (Cytiva) was pre-immobilized on the CM5 chip using standard amine coupling chemistry with a 50 μg/mL concentration. Concentrated supernatant containing hACE2-mFc or miACE2-mFc protein was captured on the chip using this immobilized antibody. Serially diluted WT RBD (12.5, 25, 50, 100, 200 nM), Alpha RBD and Mink-Y453F RBD (3.125, 6.25, 12.5, 25, 50 nM), Beta RBD, Gamma RBD, and Mink-N501T RBD (6.25, 12.5, 25, 50, 100 nM), Mink-F486L RBD (25, 50, 100, 200, 400 nM), RBD K417N and RBD K417T (20, 40, 80, 160, 320 nM), RBD N501Y/E484K (0.5, 1, 2, 4, 8 nM), RBD N501Y/K417N (4, 8, 16, 32, 64 nM), RBD N501Y/K417T (2, 4, 8, 16, 32 nM), and RBD E484K (5, 10, 20, 40, 80 nM) were flowed over the chip to evaluate hACE2 binding. Various concentrations of WT RBD (2.5, 5, 10, 20, 40 μM), Mink-Y453F RBD (40, 80, 160, 320, 640 nM), Mink-F486L RBD (160, 320, 640, 1280, 2560 nM), and Mink-N501T RBD (320, 640, 1280, 2560, 5120 nM) were also used to evaluate miACE2 binding. The chip was regenerated after each reaction using glycine (pH 1.7). The equilibrium dissociation constants (*K*_D_) of each pair of RBD-hACE2 interaction and RBD-miACE2 interaction were analyzed using a 1:1 binding model and steady state affinity model in the Biacore Insight Evaluation 2.0.15.12933 software (GE Healthcare), respectively. These results were then visualized using Graphpad Prism 8.

### Flow cytometry analysis

These assays used a stable BHK21 cell line expressing hACE2 which was first constructed. Briefly, lentiviruses were packaged in HEK293T cells co-transfected with pLV-C-GFPSpark-hACE2 (Sino Biological) and helper plasmid pLP1, pLP2, and pLP/VSV-G (Invitrogen) at a ratio of 20:20:13:5 using Lipofectamine 2000. After 72 h, the supernatants containing the hACE2 lentiviruses were collected and used to infect BHK21 cells. GFP-positive cells were selected using a BD FACSAriaIII (Becton, Dickinson and Company) and separated into 96-well cell culture plates. A single positive colony was then picked for further culture. The cells were stored in liquid nitrogen following two times of cell sorting. The hACE2-expressing cells were grown for approximately 12 passages, and incubated with Phosphate Buffer Saline (PBS) or 2 μg/mL RBD protein (including MERS-CoV RBD, SARS-CoV-2 WT RBD, Alpha RBD, Beta RBD, Gamma RBD, Mink-Y453F RBD, Mink-N501T RBD, and Mink-F486L RBD) at 37 °C for 30 min. MERS-CoV RBD was used as a negative control. After washing with PBS, the cells were stained with APC anti-His tag antibody (1:500; BioLegend) at 37 °C for 30 min. These cells were then washed and resuspended in 200 μL PBS before being evaluated using a BD FACSCanto II (Becton, Dickinson and Company). The percentage of RBD-binding cells can be described as the ratio of RBD-binding cells (Q2) to hACE2-positive cells (Q2 and Q3). Each group comprised at least three replicates and the FACS graphics were generated using FlowJo V7.6 software. Statistical analysis was performed using Graphpad Prism 8.

### Molecular dynamics (MD) simulations

Briefly, both the WT RBD-hACE2 (PDB: 6LZG) and Mink-Y453F RBD-hACE2 structures were stripped of their N-acetyl-β-glucosaminide glycans and crystal structural waters and then used in ten parallel molecular dynamics (MD) simulations with different random seeds. All MD simulations were performed using GROMACS (version 2020.5)^39^ on GPU with the CHARMM36 protein force field and TIP3P water model^40,41^. All calculations were applied using an atom-based truncation scheme and updated heuristically with a list cutoff of 12 Å, a non-bond cutoff of 12 Å, and a force switching function initiated at 10 Å for Van der Waals interactions. Long-range electrostatic interactions were computed using Particle Mesh Ewald method with fourth-order cubic interpolation and 1.6 Å grid spacing^42^. To equilibrate solvent molecules around the solute, each system was minimized using the steepest descent algorithm with a maximum force of <1000 kJ/mol/nm, followed by 200 ps of NVT MD equilibration and 200 ps of NPT equilibration with 1000 kJ/mol·nm2 harmonic positional restraints on the heavy atoms of the protein, and 200 ps NPT with 1000 kJ/mol·nm2 harmonic positional restraints on the protein backbone heavy atoms, and 200 ps NPT with reduced harmonic positional restraints of 500 kJ/mol·nm^2^ on the protein backbone heavy atoms. Each equilibrated system was finally subjected to 100 ns NPT at 300 K using a Velocity rescaling thermostat without any restraints on the protein atoms^43^. During MD simulation, the LINCS algorithm^44^ used to constrain all of the bonds which rely on hydrogen atoms using a time step of 2 fs, and water molecules were restrained using the SETTLE algorithm^45^.

### Molecular mechanics/Poisson-Boltzmann surface area (MM/PBSA) binding energy calculations

For each WT RBD-hACE2 and Mink-Y453F RBD-hACE2 complex, 100 snapshots were extracted from the last 20 ns trajectories. A total of 1000 snapshots were combined and subjected to MM/PBSA calculations using the g_mmpbsa tool in GROMACS^46^. The solute dielectric constant was set to 2 and the entropy contribution was ignored. The binding energy was calculated as ΔE_Binding_ = E_RBD-hACE2_ − (E_RBD_ + E_hACE2_). The energy components consisted of the molecular mechanics (MM) potential energy E_MM_, and the solvation energy E_solvation_.

### Production and quantification of pseudoviruses

Pseudoviruses containing SARS-CoV-2 variant S protein and the backbone of deficient vesicular stomatitis virus (VSV) vector (VSV-ΔG-GFP) (BrainVTA) were generated using the previously described protocols^47,48^. In brief, 30 μg of S plasmid with a C terminal 18 amino acids truncation was transfected into HEK293T cells cultured in a 10 cm dish, then after 24 h the VSV-ΔG-GFP pseudoviruses were added into the cell supernatant. The inoculum was then removed following incubation at 37 °C for 2 h and the cells were washed with PBS and cultured in DMEM supplemented with both 10% FBS and anti-VSV-G antibody (produced by I1Hybridoma ATCC^®^ CRL2700™). Then 20 h post-infection, the supernatants were harvested, filtered (0.45 μm filter, Millipore, Cat#SLHP033RB), aliquoted, and stored at −80 °C.

Prior to quantification, the unpackaged RNA in the SARS-CoV-2 pseudoviruses was removed using a 0.5 U/μL BaseMuncher endonuclease (Abcam) treatment at 37 °C for 1.5 h. Viral RNA was extracted using an RNA extraction kit (Bioer Technology) and quantified using a quantitative RT-PCR assay performed using a 7500 Fast Real-Time PCR system (Applied Biosystems). The primers and probe used to detect the L gene of the VSV virus are as described in the literature^49^: VSV-F: TGATACAGTACAATTATTTTGGGAC; VSV-R: GAGACTTTCTGTTACGGGATCTGG; VSV-probe: FAM-ATGATGCATGATCCWGC-TAMRA.

### Pseudovirus infection assays

The pseudovirus particles for SARS-CoV-2 and its variants were normalized and then diluted to the same amount using quantitative RT-PCR. Then 100 μL of each pseudovirus was added to the cultured Huh7 cells. 15 h later, the plates were imaged and the number of infected cells, positive for GFP, was determined using a BD FACSCanto II. Each group contained at least three replicates. Statistical analysis was performed using Graphpad Prism 8.

### Pseudovirus neutralization assays

Serially diluted hACE2 proteins (The maximum concentration was 240 μg/mL) were incubated with SARS-CoV-2 or its variants at 37 °C for 1 h. Then the mixtures were added into the Vero cells in the 96-well plates. After 15 h, the number of infected cells was analyzed using CQ1 confocal quantitative image cytometer (Yokogawa). Each group contained at least three replicates. The EC_50_ values were calculated using GraphPad Prism 8.

### Statistic of SARS-CoV-2 variants S sequences

SARS-CoV-2 S protein sequences were downloaded from the GISAID Initiative database (gisaid.org)^50^ with the accession ID of the newest sequence submitted to the GISAID Initiative at the time of evaluation being EPI_ISL_1159228. We discarded the sequences (a total of 168,190) that contain sequencing errors (including any sequences with “X” in the sequence) or inexact collection time. This left 506,858 sequences which were then individually aligned with reference Wuhan-Hu-1 S sequence (GISAID: EPI_ISL_402119) using Mafft v7.310^51^. The S protein sequences with the fewest mutations in each SARS-CoV-2 variant were selected for further statistical analysis. Based on the collection time, the cumulative weekly number of new sequences was calculated for the period between April 20, 2020 and March 1, 2021. For Mink-Y453F, Mink-F486L, and Mink-N501T, the number of new sequences isolated in humans or minks were calculated individually. The frequency was calculated as the ratio of the cumulative number of each variant to the total cumulative number of sequences.

### Reporting summary

Further information on research design is available in the Nature Research Reporting Summary linked to this article.

## Supplementary information

Supplementary Information

Reporting summary

## Data Availability

The accession numbers for the atomic coordinates and diffraction data reported in this paper are PDB: 7EKF [10.2210/pdb7EKF/pdb] (Structure of SARS-CoV-2 Alpha variant spike receptor-binding domain complexed with human ACE2), 7EKG [10.2210/pdb7EKG/pdb] (Structure of SARS-CoV-2 Beta variant spike receptor-binding domain complexed with human ACE2), 7EKC [10.2210/pdb7EKC/pdb] (Structure of SARS-CoV-2 Gamma variant spike receptor-binding domain complexed with human ACE2), 7EKH [10.2210/pdb7EKH/pdb] (Structure of SARS-CoV-2 spike receptor-binding domain Y453F mutation complexed with human ACE2) and 7EKE [10.2210/pdb7EKE/pdb] (Structure of SARS-CoV-2 spike receptor-binding domain F486L mutation complexed with human ACE2). Source data are provided with this paper.

## Source data

Source Data
