# Supplementary material for: Molecular insights into receptor binding of recent emerging SARS-CoV-2 variants
Source: Nat Commun. 2021 Oct 20;12:6103. doi: 10.1038/s41467-021-26401-w (PMC8528823; doi:10.1038/s41467-021-26401-w)
Supplement: Supplementary file 1 — Supplementary Information [file 41467_2021_26401_MOESM1_ESM.pdf]

Supplementary Information

Molecular insights into receptor binding of recent emerging SARS-CoV-2 variants

Pengcheng Han[1,2,3,†], Chao Su[1,4,†], Yanfang Zhang[1,5,†], Chongzhi Bai[6,7,†], Anqi Zheng[1,8], Chengpeng Qiao[1], Qing Wang[9], Sheng Niu[1,10], Qian Chen[1,11], Yuqin Zhang[1,8], Weiwei Li[1,8], Hanyi Liao[1,8], Jing Li[1], Zengyuan Zhang[1,8], Heecheol Cho[2], Mengsu Yang[4], Xiaoyu Rong[1,12], Yu Hu[1,13], Niu Huang[9,14], Jinghua Yan[15], Qihui Wang[1], Xin Zhao[1,16,*], George Fu Gao[1,4,8,*], Jianxun Qi[1,8,*]

[*]These authors contributed equally
[†]Corresponding author. Email: jxqi@im.ac.cn (J.X.); gaof@im.ac.cn (G.F.G); zhaoxin@im.ac.cn (X.Z.)

Supplementary Table 1. Data collection and refinement statistics

	Alpha RBD-hACE2	Beta RBD-hACE2	Gamma RBD-hACE2	Mink-Y453F RBD-hACE2	Mink-F486L RBD-hACE2
**Data collection**					
Space group	*P41212*	*P41212*	*P41212*	*P41212*	*P41212*
Cell dimensions					
a, *b*, *c* (Å)	104.53, 104.53, 230.47	105.15, 105.15, 230.94	103.354, 103.354, 229.784	103.354, 103.354, 229.784	104.316, 104.316, 228.37
α, β, γ (°)	90, 90, 90	90, 90, 90	90, 90, 90	90, 90, 90	90, 90, 90
Resolution (Å)	28.12-2.85 (2.92-2.85)[a]	27.01-2.63 (2.70-2.63)[a]	50.00-2.80 (2.90-2.80)[a]	50.00-2.40 (2.49-2.40)[a]	50.00-2.70 (2.80-2.70)[a]
Unique reflections	30710	39380	31516	49236	35606
R_{merge} [b]	0.187 (2.105)[a]	0.125 (2.236)[a]	0.141 (1.381)[a]	0.177 (1.916)[a]	0.178 (1.514)[a]
R_{pim} [c]	0.041 (0.444)[a]	0.027 (0.476)[a]	0.043 (0.421)[a]	0.041 (0.508)[a]	0.042 (0.351)[a]
$I/\sigma I$	16.5 (2.1)[a]	21.9 (2.0)[a]	17.884 (1.726)[a]	19.826 (1.661)[a]	20.354 (2.388)[a]
$CC_{1/2}$	0.999 (0.684)[a]	1.000 (0.744)[a]	0.997 (0.717)[a]	0.998 (0.565)[a]	0.996 (0.771)[a]
Completeness (%)	99.9 (100)[a]	99.0 (100)[a]	99.9 (100)[a]	99.8 (98.5)[a]	100 (100)[a]
Redundancy	21.7 (22.8)[a]	21.8 (22.6)[a]	11.3(11.6)[a]	18.7 (14.6)[a]	19.0 (18.2)[a]
**Refinement**					
Resolution (Å)	27.86-2.85	26.91-2.63	19.92-2.80	20.39-2.40	20.50-2.70
No. reflections	30617	39271	29219	46716	34993
R_{work} / R_{free} [c]	0.2081/0.2238	0.2138/0.2484	0.2141/0.2252	0.2098/0.2280	0.2119/0.2407
No. atoms					
Protein	6436	6414	6420	6410	6408
Ligand/ion	85	99	99	57	57
Water	0	21	50	250	0
B-factors					
Protein	72.44	72.49	52.98	45.51	45.31
Ligand/ion	116.23	119.70	104.26	85.01	90.45

Water		59.42	38.55	40.79	
R.m.s. deviations					
Bond lengths (Å)	0.005	0.004	0.002	0.005	0.005
Bond angles (°)	0.695	0.621	0.477	0.742	0.691
Ramachandran plot					
Favored (%)	98.22	97.97	98.48	97.59	97.59
Allowed (%)	1.78	2.03	1.52	2.41	2.41
Outliers (%)	0.00	0.00	0.00	0.00	0.00

[a] Values in parentheses are for highest-resolution shell.

Supplementary Table 2. MM/PBSA calculation results of WT RBD-hACE2 and Mink-Y453F RBD-hACE2 systems

	ΔE_{MM} (kcal·mol^{-1})	$\Delta E_{Solvation}$ (kcal·mol^{-1})	$\Delta E_{Binding}$ (kcal·mol^{-1})
Y453F	-396.36 ± 21.94	150.04 ± 37.50	-246.33 ± 36.30
RBD	-387.28 ± 21.69	148.85 ± 36.40	-238.43 ± 36.06
Δ (Y453F-WT)	-9.08	1.19	-7.90

MM/PBSA: molecular mechanics/Poisson-Boltzmann surface area; Y453F: Mink-Y453F RBD-hACE2; WT: WT RBD-hACE2

Supplementary Table 3. Energy decomposition of the single residues in the RBD-ACE2 binding interface

Residue	$\Delta\Delta E_{MM}$ ($kcal \cdot mol^{-1}$)	$\Delta\Delta E_{Solvation}$ ($kcal \cdot mol^{-1}$)	$\Delta\Delta E_{Binding}$ ($kcal \cdot mol^{-1}$)
hACE2 D30	2.96	-3.99	-1.03
RBD R403	-1.08	-0.23	-1.31
RBD Y453F	0.48	-1.51	-1.03
RBD K417	-0.12	-0.37	-0.50

The binding energies are analyzed at a cutoff of -0.5 $kcal \cdot mol^{-1}$ ($\Delta\Delta E = \Delta E_{Y453F} - \Delta E_{WT}$).

Y453F: Mink-Y453F RBD-hACE2; WT: WT RBD-hACE2.

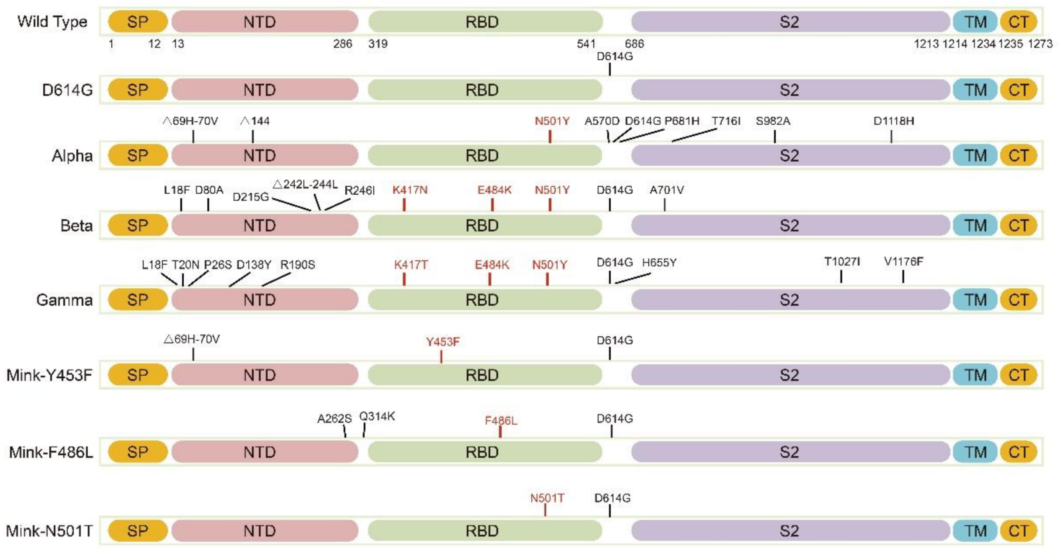

Supplementary Figure 1. Schematic representation of SARS-CoV-2 wild type (WT) and variant S proteins. The mutations of amino acids and positions are as indicated. SP: signal peptide, NTD: N-terminal domain, RBD: receptor-binding domain, TM: transmembrane, CT: C-terminal cytoplasmic tail domain.

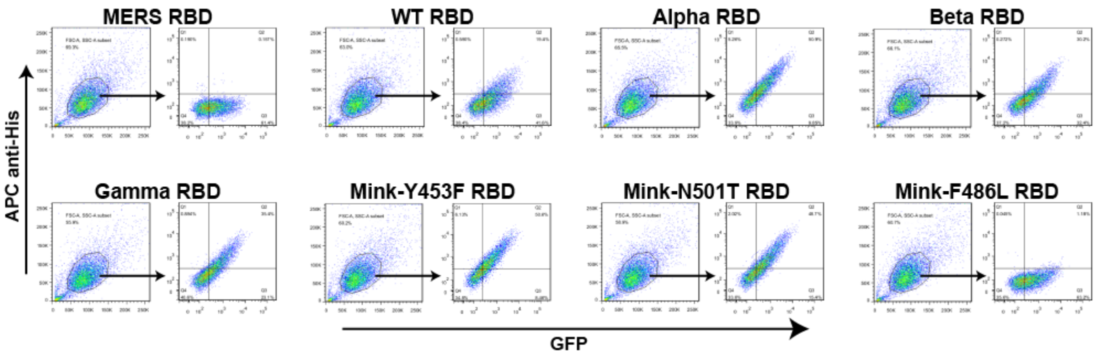

Supplementary Figure 2. The gating strategy for flow cytometry analysis of SARSCoV-2 variants RBDs binding to BHK21 cells expressing hACE2. The cells expressing hACE2-GFP were incubated with His-tagged MERS-CoV RBD, SARS-CoV-2 WT RBD, Alpha RBD, Beta RBD, Gamma RBD, Mink-Y453F RBD, Mink-N501T RBD, and Mink-F486L RBD, respectively, and then were the strained with the anti-His/APC antibody. The cells populated based on the forward scatter (FSC) and the side scatter (SSC) signals were first gated with the GFP fluorescent densities. The GFP$^+$ cell population were further divided into the APC$^+$ population (cells binding to RBD) and APC$^-$ population (cells not binding to RBD).

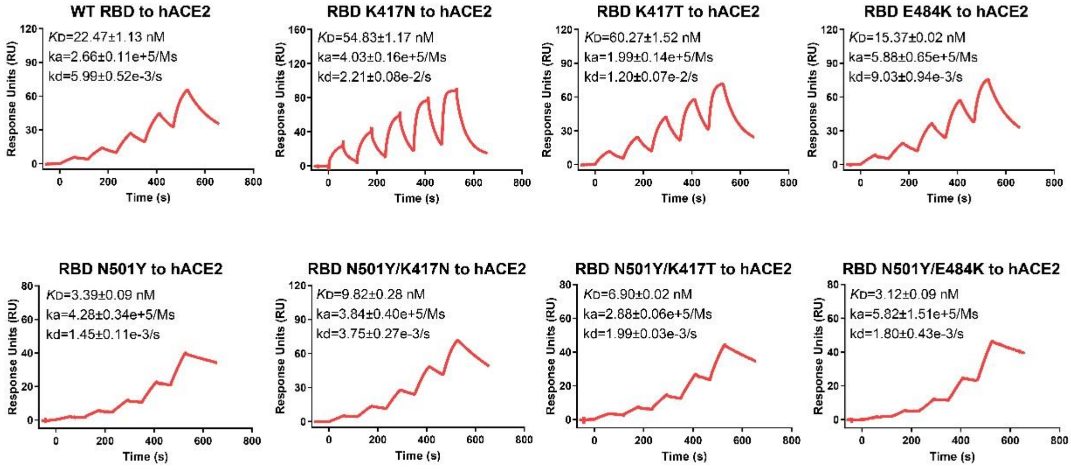

Supplementary Figure. 3 Binding affinity of SARS-CoV-2 RBD mutations to ACE2, characterized by SPR. Mouse Fc (mFc)-fused hACE2 in the supernatant was captured in the CM5 chip *via* its interaction with the pre-immobilized anti-mFc antibody. Various concentrations of SARS-CoV-2 WT RBD, RBD K417N, RBD K417T, RBD E484K, RBD N501Y, RBD N501Y/K417N, RBD N501Y/K417T, and RBD N501Y/E484K protein were used to evaluate their binding affinity for hACE2. K_D, ka, and kd values are all recorded and the representative results from three experiments are shown. The data are presented as the mean ± SEM of three independent replicates (n=3).

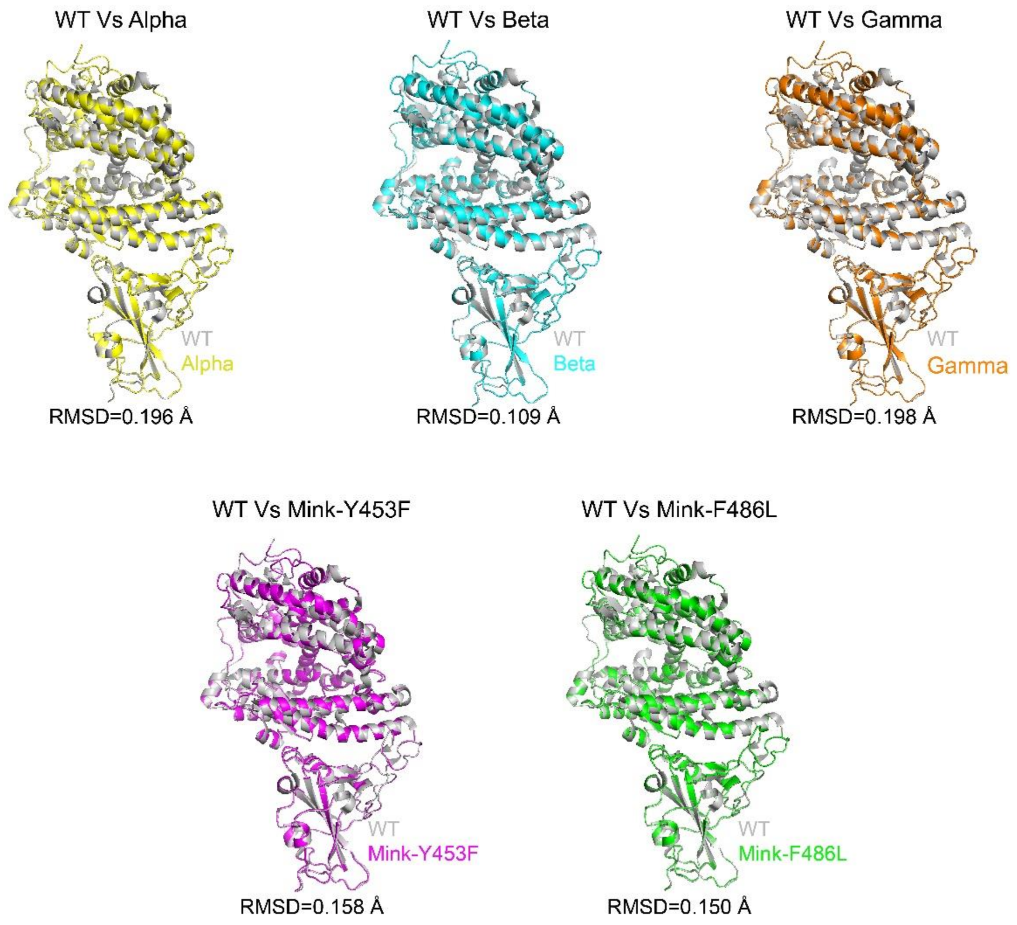

Supplementary Figure 4. Overall structural comparison of each variant RBD-hACE2 with the WT RBD-hACE2. The structures of Alpha RBD-hACE2, Beta RBD-hACE2, Gamma RBD-hACE2, Mink-Y453F RBD-hACE2, and Mink-F486L RBD-hACE2 were all aligned with WT RBD-hACE2 (PDB: 6LZG) and are highlighted in yellow, cyan, orange, magenta, and green, respectively. WT RBD-hACE2 is in gray. Root-mean-square deviation (RMSD) is shown.

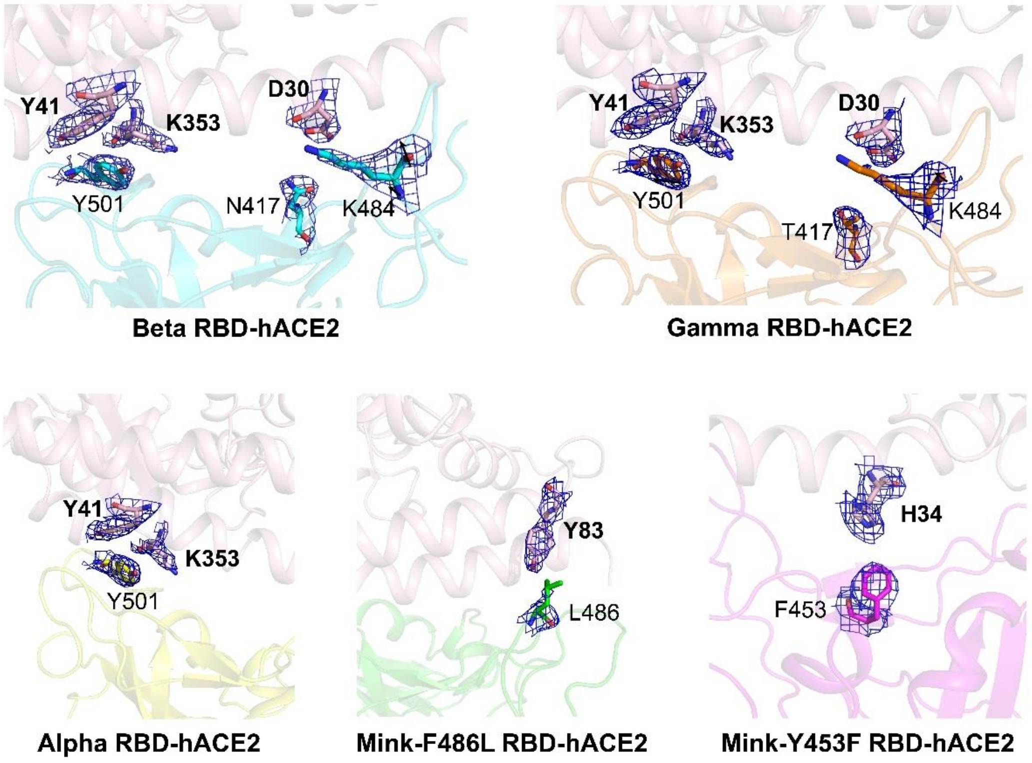

Supplementary Figure 5. Representative electron density maps for each of the five complex structures evaluated in this study. The final 2Fo-Fc density maps of the complex structures for hACE2 bound to Beta RBD, Gamma RBD, Alpha RBD, Mink-F486L RBD, and Mink-Y453F RBD are drawn in blue mesh contoured at 1 sigma, respectively.

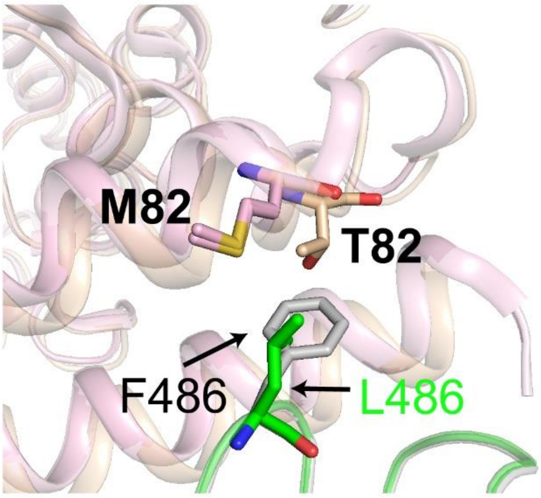

Supplementary Figure 6. Structural comparison of miACE2 with both Mink-F486L RBD-hACE2 and WT RBD-hACE2 (PDB: 6LZG). The structure of miACE2 was predicted using SWISS-MODE (swissmodel.expasy.org) with the previously reported structures of ACE2-B0AT1 complex (PDB: 6M18) as a template. miACE2, hACE2, SARS-CoV-2 WT RBD, and Mink-F486L RBD are colored in wheat, light pink, gray and green, respectively. hACE2 M82, miACE2 T82, WT RBD F486, and Mink-F486L RBD L486 are as indicated.

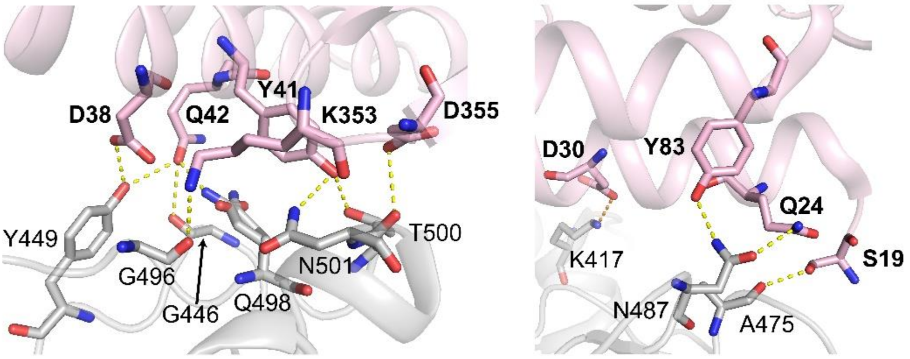

Supplementary Figure 7. Detailed hydrogen bond interaction between SARS-CoV-2 WT RBD and hACE2. Hydrogen bond interactions in the structure of SARS-CoV-2 WT RBD-hACE2 (PDB: 6LZG) were analyzed at a cutoff of 3.5 Å. hACE2 and SARS-CoV-2 WT RBD are in light pink and gray, respectively. The key residues are shown with stick and are labeled.

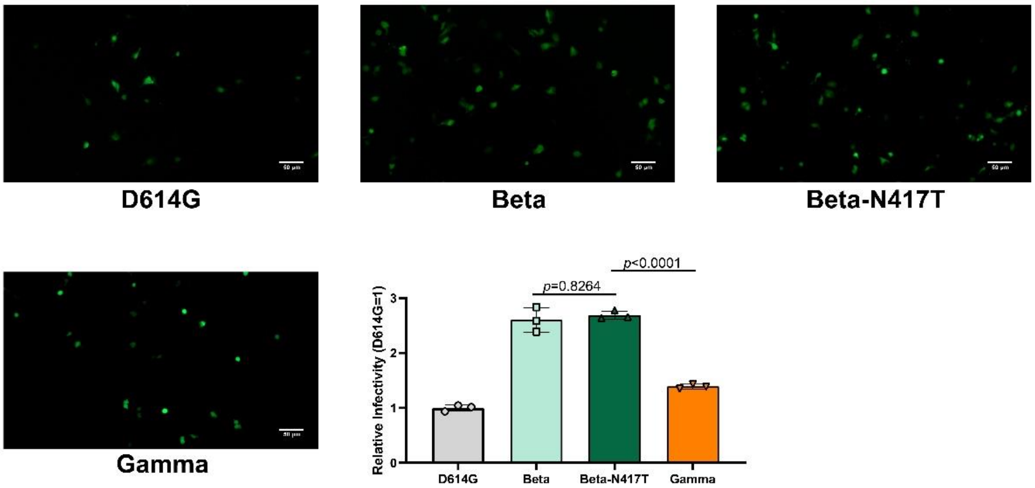

Supplementary Figure 8. Entry of SARS-CoV-2 variant pseudoviruses into Huh7 cells. The SARS-CoV-2 D614G, Beta, Beta-N417T, and Gamma pseudoviruses' entry into Huh7 cells as evidenced by GFP expression in transduced cells. The GFP-positive cells were quantified using FACS and representative results from three experiments are shown. The values indicate the mean of the three experiments and the bar suggest the SD. Relative infectivity was normalized against that of the D614G pseudovirus. Statistical significance was analyzed using a one-way ANOVA with Tukey’s multiple comparison test for multiple groups.

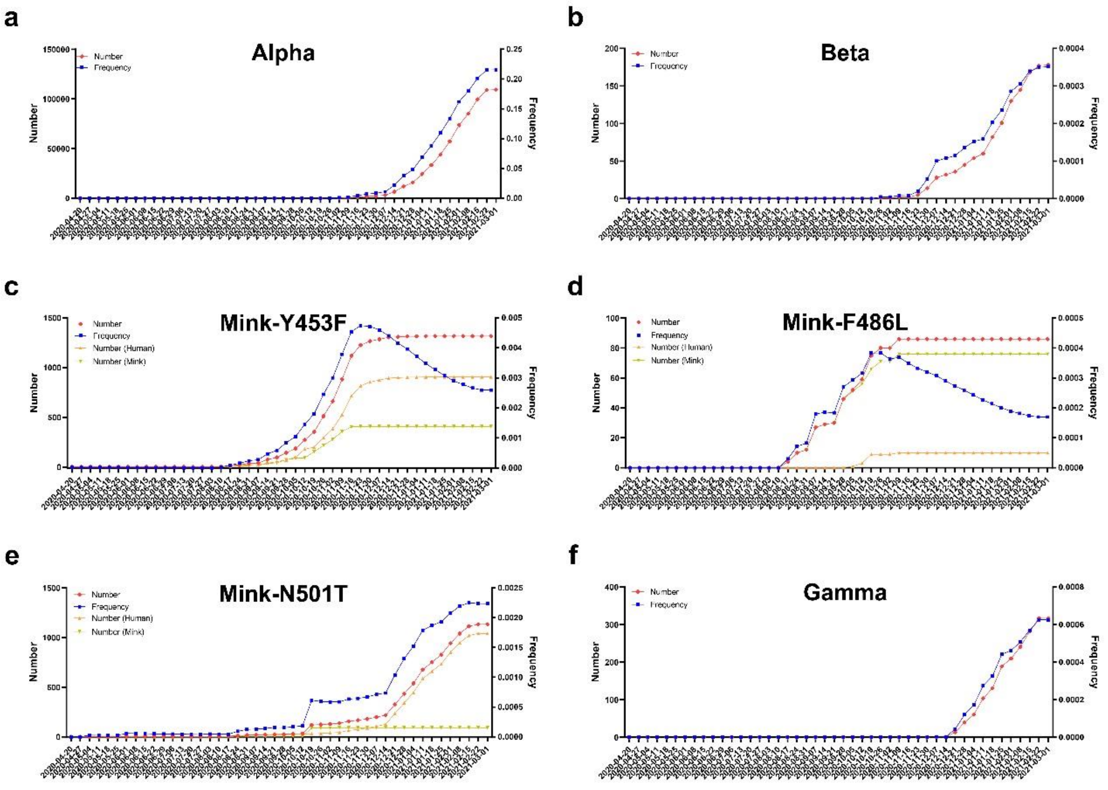

Supplementary Figure 9. Number and frequency of SARS-CoV-2 variant S sequences in the GISAID Initiative database. a-**f** The cumulative weekly number and frequency of S sequence variations for Alpha (**a**), Beta (**b**), Mink-Y453F (**c**), Mink-F486L (**d**), Mink-N501T (**e**), and Gamma (**f**) were calculated between April 20, 2020 and March 1, 2021. For Mink-N501T, Mink-F486L, and Mink-Y453F, the number of new sequences isolated in humans or minks were also calculated.
