# Supplementary material for: Molecular insights into receptor binding of recent emerging SARS-CoV-2 variants
Source: Nat Commun. 2021 Oct 20;12:6103. doi: 10.1038/s41467-021-26401-w (PMC8528823; doi:10.1038/s41467-021-26401-w)
Supplement: Supplementary file 2 — Reporting summary [file 41467_2021_26401_MOESM2_ESM.pdf]

nature portfolio

Corresponding author(s): George F.Gao

Last updated by author(s): Sep 16, 2021

Reporting Summary

Nature Portfolio wishes to improve the reproducibility of the work that we publish. This form provides structure for consistency and transparency in reporting. For further information on Nature Portfolio policies, see our Editorial Policies and the Editorial Policy Checklist.

Statistics

For all statistical analyses, confirm that the following items are present in the figure legend, table legend, main text, or Methods section.

n/a	Confirmed	
☐	☒	The exact sample size (*n*) for each experimental group/condition, given as a discrete number and unit of measurement
☐	☒	A statement on whether measurements were taken from distinct samples or whether the same sample was measured repeatedly
☐	☒	The statistical test(s) used AND whether they are one- or two-sided<br>*Only common tests should be described solely by name; describe more complex techniques in the Methods section.*
☒	☐	A description of all covariates tested
☒	☐	A description of any assumptions or corrections, such as tests of normality and adjustment for multiple comparisons
☐	☒	A full description of the statistical parameters including central tendency (e.g. means) or other basic estimates (e.g. regression coefficient) AND variation (e.g. standard deviation) or associated estimates of uncertainty (e.g. confidence intervals)
☐	☒	For null hypothesis testing, the test statistic (e.g. *F*, *t*, *r*) with confidence intervals, effect sizes, degrees of freedom and *P* value noted<br>*Give P values as exact values whenever suitable.*
☒	☐	For Bayesian analysis, information on the choice of priors and Markov chain Monte Carlo settings
☒	☐	For hierarchical and complex designs, identification of the appropriate level for tests and full reporting of outcomes
☒	☐	Estimates of effect sizes (e.g. Cohen's *d*, Pearson's *r*), indicating how they were calculated

Our web collection on statistics for biologists contains articles on many of the points above.

Software and code

Policy information about availability of computer code

Data collection	Shanghai Synchrotron Radiation Facility (SSRF) BL19U, BD FACSCanto II, BD FACSAriaIII, CQ1 confocal quantitative image cytometer
Data analysis	Phenix 1.19, Phaser, CCP4 7.1, HKL2000, Coot 0.8.9, MolProbity, PyMOL 4.5, GraphPad Prism 8.0, Biacore Insight Evaluation 2.0.15.12933, GROMACS 2020.5, Mafft 7.310, FlowJo 7.6

For manuscripts utilizing custom algorithms or software that are central to the research but not yet described in published literature, software must be made available to editors and reviewers. We strongly encourage code deposition in a community repository (e.g. GitHub). See the Nature Portfolio guidelines for submitting code & software for further information.

Data

Policy information about availability of data

All manuscripts must include a data availability statement. This statement should provide the following information, where applicable:

- Accession codes, unique identifiers, or web links for publicly available datasets
- A description of any restrictions on data availability
- For clinical datasets or third party data, please ensure that the statement adheres to our policy

The structural data that support the findings of this study have been deposited in the Protein Data Bank. The accession numbers for the atomic coordinates and diffraction data reported in this paper are PDB: 7EKF (Structure of SARS-CoV-2 Alpha variant spike receptor-binding domain complexed with human ACE2), 7EKG (Structure of SARS-CoV-2 Beta variant spike receptor-binding domain complexed with human ACE2), 7EKC (Structure of SARS-CoV-2 Gamma variant spike receptor-binding domain complexed with human ACE2), 7EKH (Structure of SARS-CoV-2 spike receptor-binding domain Y453F mutation complexed with human ACE2) and 7EKE (Structure of SARS-CoV-2 spike receptor-binding domain F486L mutation complexed with human ACE2).

Field-specific reporting

Please select the one below that is the best fit for your research. If you are not sure, read the appropriate sections before making your selection.

☒ Life sciences ☐ Behavioural & social sciences ☐ Ecological, evolutionary & environmental sciences

For a reference copy of the document with all sections, see nature.com/documents/nr-reporting-summary-flat.pdf

Life sciences study design

All studies must disclose on these points even when the disclosure is negative.

Sample size	SPR experiments, FACS experiment, pseudovirus infection assays, and pseudovirus neutralization assays were repeated three times in this study because it is common in the biological experiment. No statistical methods were used to predetermine sample size.
Data exclusions	No data were excluded from the analysis.
Replication	Experiments were repeated at least to n=3 to verify reproducibility. All attempts at replication were successful.
Randomization	For SPR experiments, FACS experiment, pseudovirus infection assays, and pseudovirus neutralization assays, the samples were allocated in different sequence.
Blinding	For SPR experiments, FACS experiment, pseudovirus infection assays, and pseudovirus neutralization assays, when collecting the data, investigators were blinded to the group. Because samples were marked as numbers, only after checking the sheet, the group allocation were known. For other experiments, the investigators were not blinded to group allocation during data collection or analysis. Because blinding has no effect on the experimental results.

Reporting for specific materials, systems and methods

We require information from authors about some types of materials, experimental systems and methods used in many studies. Here, indicate whether each material, system or method listed is relevant to your study. If you are not sure if a list item applies to your research, read the appropriate section before selecting a response.

Materials & experimental systems

n/a	Involved in the study	
☐	☒	Antibodies
☐	☒	Eukaryotic cell lines
☒	☐	Palaeontology and archaeology
☒	☐	Animals and other organisms
☒	☐	Human research participants
☒	☐	Clinical data
☒	☐	Dual use research of concern

Methods

n/a	Involved in the study	
☒	☐	ChIP-seq
☐	☒	Flow cytometry
☒	☐	MRI-based neuroimaging

Antibodies

Antibodies used	APC anti-His Tag antibody was from BioLegend (Cat# 362605; RRID: AB_2751870), anti-mIgG antibody was from Cytiva (Cat# BR100838)
Validation	All antibodies were verified by the supplier and each lot has been quality tested. All the antibodies used are from commercial sources and have been validated by the vendors. Validation data are available on the manufacturer's website.<br>APC anti-His Tag antibody (Cat# 362605): https://www.biolegend.com/en-us/products/apc-anti-his-tag-antibody-14783.<br>anti-mIgG antibody (Cat# BR100838): https://www.cytivalifesciences.com/en/us/shop/protein-analysis/spr-label-free-analysis/spr-consumables/capture-reagents/mouse-antibody-capture-kit-p-05986

Eukaryotic cell lines

Policy information about cell lines

Cell line source(s)	Sf9 and High Five insect cell line were from Invitrogen; BHK cells and HEK293T cells (ATCC CRL-3216) were from ATCC; Epxi293F cells were from Gibco; Huh7 cells were from Institute of Basic Medical Sciences CAMS.
Authentication	No cell authentication was used.
Mycoplasma contamination	All cell lines tested negative for mycoplasma.

Commonly misidentified lines (See ICLAC register)	No misidentified lines were used.

Flow Cytometry

Plots

Confirm that:

- [x] The axis labels state the marker and fluorochrome used (e.g. CD4-FITC).
- [x] The axis scales are clearly visible. Include numbers along axes only for bottom left plot of group (a 'group' is an analysis of identical markers).
- [x] All plots are contour plots with outliers or pseudocolor plots.
- [x] A numerical value for number of cells or percentage (with statistics) is provided.

Methodology

Sample preparation	The description is stated in Methods section. Stable hACE2-expressed BHK21 cells were incubated with 2 μg/mL RBD protein and then stained with APC anti-His tag antibody. Huh7 cells were added by 100 μL of pseudovirus.
Instrument	BD FACSCanto II, BD FACSAriaIII
Software	BDFACSDiva software was used to collect data, while FlowJo was used for data analysis.
Cell population abundance	The instrument counts 10,000 or 50,000 cells autonomously.
Gating strategy	Positive populations were defined using not stained cells as reference.

- [x] Tick this box to confirm that a figure exemplifying the gating strategy is provided in the Supplementary Information.
